# Mineral and Bone Consequences of High Dose Denosumab Therapy to Treat an Aneurysmal Bone Cyst, a Child Case Report

**DOI:** 10.3389/fendo.2021.698963

**Published:** 2021-07-16

**Authors:** Giulia Del Sindaco, Pablo Berlanga, Laurence Brugières, Eric Thebault, Giovanna Mantovani, Philippe Wicart, Agnès Linglart

**Affiliations:** ^1^ Endocrinology Unit, Fondazione IRCCS Ca’ Granda Ospedale Maggiore Policlinico, ERN BOND, Milan, Italy; ^2^ Department of Clinical Sciences and Community Health, University of Milan, Milan, Italy; ^3^ AP-HP, Service d’endocrinologie et diabète de l’enfant, ERN BOND, ERN for rare endocrine disorders, et Plateforme d’expertise des maladies rares, Hôpital Bicêtre Paris Saclay, Le Kremlin-Bicêtre, France; ^4^ Department of Pediatric and Adolescent Oncology, Gustave Roussy Cancer Center, Paris-Saclay University, Villejuif, France; ^5^ AP-HP, Centre de Référence des maladies rares du métabolisme du Calcium et du Phosphate, filière OSCAR, Paris, France; ^6^ AP-HP, Department of Pediatric Orthopedic Surgery, Necker - Enfants Malades University Hospital, Paris, France. Paris Descartes University, Paris, France; ^7^ Université de Paris Saclay, INSERM, U1185, Le Kremlin-Bicêtre, France

**Keywords:** denosumab, aneurysmal bone cyst, hypercalcemia, bisphosphonate, bone modeling

## Abstract

Aneurysmal bone cysts (ABCs) are rare benign pseudotumoral bone lesions with potential aggressive behavior due to the extensive destruction of surrounding bone. Traditionally, these tumors were treated with open surgery, but there is more and more a shift to less invasive procedures. In particular, treatment for spinal ABCs is generally unsatisfactory due to the risk of morbidity, neurological impairment and recurrence, and there is a need for innovative therapies. Denosumab has been reported as a useful treatment in giant cell tumors of bone (GCTB), so its efficacy has been tested also in other fibro-osseus lesions affecting children and adolescents, such as spinal aneurysmal bone cysts. The pediatric literature is limited to case reports and small series, all of which highlight the efficacy of this treatment on lesions growth and associated bone pain. Some of these reports have already reported well known side effects associated with denosumab, such as hypocalcemia at the beginning of the treatment, and rebound hypercalcemia at the discontinuation. The latter seems to be more frequent in children and adolescents than in adults, probably due to the higher baseline bone turnover in children. In addition, the use of denosumab in young patients could affect both bone modeling and remodeling, even if the consequences on the growing skeleton have not been reported in detail. Here we describe the case of a spinal ABC diagnosed in an 8-year old young boy which was not accessible to surgery but responded favorably to denosumab. Our aim is to describe the rapid changes in mineral and bone homeostasis in this patient, that required advice from the experts of the European Reference Network (ERN) for rare bone and endocrine diseases.

## Introduction

Aneurysmal bone cysts (ABCs) are rare tumors accounting for 1-2% of the primary bone tumors and for 15% of primary spine tumors (1). ABCs arising from the mobile spine account for the 10-30% of all ABC cases and manifest with axial pain as the most common symptom (2).

Although ABCs are benign pseudotumoral bone lesions, they may have potentially aggressive behavior due to the extensive destruction of surrounding bone (3). ABCs belong to a family of benign (myo) fibroblastic tumor subtypes characterized by *USP6*-genetic rearrangements. These rearrangements are detected in over 60% of ABCs leading to the transcriptional upregulation of USP6, a deubiquitinating enzyme family protein. The increased expression of USP6 leads to the activation of the receptor activator of nuclear factor kappa B (NF-κB) (RANK) signaling pathway and the increased production of matrix metalloproteinase (4, 5).

ABCs contain cell types usually found in bone, including osteoclast-like multinucleated cells that express high levels of RANK, and neoplastic stromal cells that express high levels of RANK ligand (RANKL). Bone resorption and osteolysis are accrued as consequences of the RANK-RANKL increased signaling (6).

Traditionally, these tumors were treated with open surgery, but currently there is a shift to less invasive procedures mainly in specific sites with high surgical morbidity (such as spinal or vertebral tumors) or in case of tumor recurrence. In addition, treatment for spinal ABCs is still generally unsatisfactory in many cases due to the considerable risk of morbidity, neurological impairment and recurrence, and there is therefore a need for innovative and non-invasive therapies (7). Therapeutic alternatives have been reported with various efficacy and/or safety including percutaneous surgery, embolization, sclerotherapy and radiotherapy. Lastly, non-invasive treatment with denosumab or bisphosphonates has been reported to be effective in the management of the disease (8).

Denosumab is a fully human monoclonal antibody of the IgG2 immunoglobulin isotype to the receptor activator of nuclear factor-κB ligand (RANKL). It binds with high affinity and specificity to RANKL, mimicking the inhibitory effects of osteoprotegerin (OPG) on the RANK-RANKL signaling cascade and resulting in rapid suppression of bone resorption (9). The immunohistochemical similarities with giant cell tumor of bone (GCTB), lesion that display a satisfactory response to denosumab when surgery is not possible, suggest that denosumab may have positive effects on ABCs (7).

The reports on the use of denosumab for ABCs in children are currently limited to case reports and small series, highlighting mainly the beneficial effects on lesion growth, associated bone pain and facilitation of subsequent surgeries (9, 10). Some of these reports have already described well known side effects associated with denosumab, such as hypocalcemia when treatment is initiated, and rebound hypercalcemia at the treatment discontinuation (11–14).

Here we describe the mineral and bone effects of high doses of denosumab over a 5-year follow-up in a young boy who was diagnosed at the age of 8 years with a large ABC of the spine. Because of the size of the lesion and the encagement of vertebral artery, surgery was contraindicated. Medical approach was preferred in order to minimize the risk of bleeding and major complications. Therefore, denosumab was administered with optimal control of tumor size but on the other side, the onset of bone and mineral effects.

## Case Illustration

**Figure 1:**
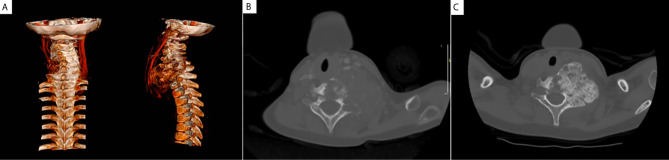
Aneurysmal bone cyst at diagnosis. MRI with 3D reconstruction at diagnosis **(A)** showing the calcified lytic lesion originating from C4-C7 hemivertebrae and encaging the vertebral artery, preventing invasive surgery; CT at diagnosis **(B)** identified a bulky lesion originating from C4-C7 hemivertebrae. CT performed 6 months after the introduction of denosumab **(C)** showed a massive calcification of the lesion that remained stable in size.

An 8-year-old male with a 6-months history of progressive growth of cervical lesion was referred to a tertiary center of Pediatric Neurosurgery complaining about progressive weakness on left arm and painful restriction of cervical movements. On admission, physical examination revealed a calcified lateral-cervical lesion of about 6 cm, not painful to the touch and mild amyotrophy of proximal left arm. Neurological examination showed a grade 2/5 muscle strength of the left triceps and a grade 4/5 muscle strength of the biceps bilaterally. No sensory deficits were detected, but biceps reflex was absent. Left deltoid paralysis was also observed, with limitation of abduction, external rotation and anteropulsion of left shoulder. Magnetic resonance imaging (**Figure 1A**) and computerized tomography (**Figure 1B**) of cervical and thoracic spine were performed and identified a bulky, cystic, calcified, lytic lesion originating from C4-C7 hemivertebrae. The lesion extended to the left epidural space and to the subclavicular region; vertebral artery was encaged. A C6 vertebral fracture was also identified. Histology confirmed then the diagnosis of aneurysmal bone cyst (ABC).

Taking account of the size of the tumor and the vascular involvement, the lesion was considered as non resectable and medical therapy with denosumab was considered after discussion in a multidisciplinary tumor board.

### Denosumab Administration and Anti-Tumoral Effect

Denosumab 70 mg/m^2^ was administered subcutaneously weekly for 4 weeks, then the maintenance dose was fixed at 1 administration monthly for one year.

After 5 doses of denosumab, neurological deficits improved with complete normalization after 4 months of treatment. CT was performed and showed a massive calcification of the lesion that remained stable in size (**Figure 1C**). Thus, denosumab was stopped, on the basis of the optimal clinical response and lack of accurate guidelines on treatment duration. The patient was then followed with tumor radiological evaluation every 3 months. Tumor evaluation performed 9 months after the end of denosumab showed tumor growth recurrence, while patient was this time asymptomatic, and denosumab was resumed. After one year of treatment, denosumab was progressively tapered firstly one administration every 2 months for 4 months, then once every 3 months during 12 months. The anti-tumoral effect was considered satisfactory as the lytic cervical lesion was stable on MRI. At physical examination the lesion was described as stable, polylobulate and calcified, measuring ~6 x 7 cm. Two and a half years after this second course of denosumab, i.e. 4.5 years after the diagnosis, denosumab treatment was stopped because of the long-term tumoral control. At last follow-up (Dec 2020), 15 months after the last injection of denosumab, the patient continues asymptomatic and tumor size is stable. The anti-tumoral efficacy of this treatment in this patient was extensively reported in a case series (15).

### Mineral Homeostasis During Denosumab Therapy

Denosumab therapy led to optimal control of symptoms and persistent radiological response yet flawed severely the mineral homeostasis.

Six months after the discontinuation of the first course of denosumab, the patient developed severe rebound hypercalcemia (total serum calcium 3.7 mmol/l, normal range 2.2-2.7 mmol/l; PTH 1 ng/l, normal range 14-74 ng/l), that required administration of a loop diuretic (furosemide) and intravenous infusion of zoledronic acid to restore normal serum calcium values. During this episode of hypercalcemia, a hypertensive crisis occurred that required the transient administration of calcium channel blockers to restore normal blood pressure.

A new episode of hypercalcemia occurred after denosumab reintroduction, this time while on denosumab tapering, 3 months after the last denosumab injection, when he was hospitalized for intense abdominal pain and loss of appetite. Blood exams revealed severe hypercalcemia (total serum calcium 3.51 mmol/l; ionized serum calcium 1.71 mmol/l), severe dehydration and acute kidney injury, but neither ECG abnormalities nor neurological complications were detected. The patient was treated with aggressive intravenous rehydration and a loop diuretic (furosemide) to progressively lower serum calcium levels; in addition, he received his dose of denosumab as initially planned. Serum calcium lowered to 2.13 mmol/l; the last dose of denosumab was administered two months after this episode. Nevertheless, 45 days after the last injection of denosumab, the patient developed another episode of severe hypercalcemia (total serum calcium 3.15 mmol/l), that was treated with intravenous zoledronic acid (see **Table 1**). Because of the rebound hypercalcemia and the abnormal bone modelling (see below), the case was presented at a European expert panel through the Clinical Patient Management System (CPMS), a web solution designed to support European Reference Networks (ERN) in improving the diagnosis and treatment of patients affected with rare diseases. Parents gave their consent and experts from the endoERN and from the rare bone disease ERN (BOND) provided their advice.

**Table 1:** Blood exams results during denosumab treatment.

Age	10 Y 1 M	10Y 7M	10Y 9M	11Y10M	12Y 2M	12Y 4M	12Y 5M	12Y 6M	12Y 8M	12Y 9M	12Y10M	12Y 11M	13Y 1M	13Y6M	13Y7M	13Y11M	14Y
Ur Creatinine (mmol/L)			7.5	17.2			3.9	17.6		6.62		8.1	7.2	21.8	6.9		
Ur Calcium (mmol/L)			**<0.2**	0.58			4.77	2.4		8.13		6.11	8.56	1.85	3.0		
Ur Ca/Crea ratio			**<0.2**	**0.03**			1.22	**0.13**		1.22		0.75	1.8	**0.09**	0.43		
Ur Phosphate (mmol/L)				38.8			16.6	60		13		7.4	11.4	34.8	7.9		
Ur DPD/Crea			**7**					**12.4**									
Creatinine (μmol/L)				41			**65**	38			36	40			41		45
Calcium (mmol/L)	**3.7**	2.23	2.33	2.49	**2.76**	**2.98**	**3.51**	2.26	**3.15**	**2.74**	2.27	2.59	2.66	2.51	2.6	2.5	2.48
Phosphate (mmol/L)		0.93	0.97	1.34			2.05	1.07		1.83	0.94	1.86	1.58	1.77	1.7	1.69	1.59
Magnesium (mmol/L)			0.91	0.94						0.79	0.86	0.78	0.77	0.80	0.9		0.8
ALP (U/L)				98				85		148		145	237	270	253		235
C-telopeptide (ng/mL) (10-14 yo: 0.70-3.10)			**0.09**					**0.33**						2.060	**3.9**		2.5
FGF23 (23.2-95.3)			43.9														
Osteocalcin (ng/ml) (4.00-60.00)			43					46					**141**		**299**		**217**
PTH (ng/l)	**1**	**236**	**180**	57			**1**	66		6.9	**102**	13	11	18.1	21	20	32
25OHvitD (ng/mL)		**17**	**20**	30			79.1	46		56	**26**	35	38	39	36	36	**28**
1,25OHvitD (ng/mL)										21				65.7			
		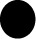					▲			▲		** 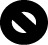 **
																	
	**6 months since discontinuation of first course of Dmab**	**Dmab 1/wk**	**Dmab 1/mo**	**Dmab** **/2mo**			**Dmab** **/3mo**	**Dmab** **/2mo**	**Zoled** **0.05** **mg/kg**	**Dmab** **/2mo**	**Dmab** **/2mo**	**Dmab** **/2mo**	**Zoled/** **6 mo**	**Zoled/** **6 mo**	**Zoled/** **6 mo**	**5 mo since last zoled**	**Zoled/** **6 mo**

Dmab, denosumab; Zoled, zoledronic acid (0,05 mg/kg); Y, year; M, months; mo, months; wk, week; 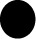 reintroduction of denosumab for the second course of treatment, ▲ denosumab injection a few days before the serum calcium measurement, 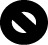 discontinuation of denosumab. Abnormal results are shown in bold.

At this point, the tumor was considered as stable, denosumab was stopped and 6 monthly IV zoledronic acid infusions were initiated in order to control the rebound hypercalcemia post-denosumab therapy. At this day, four infusions have been performed at the dose of 0.05 mg/kg (last infusion Jan 2021), the patient is asymptomatic, and the calcium levels remained below the upper limit of normal.

**Figure 2:**
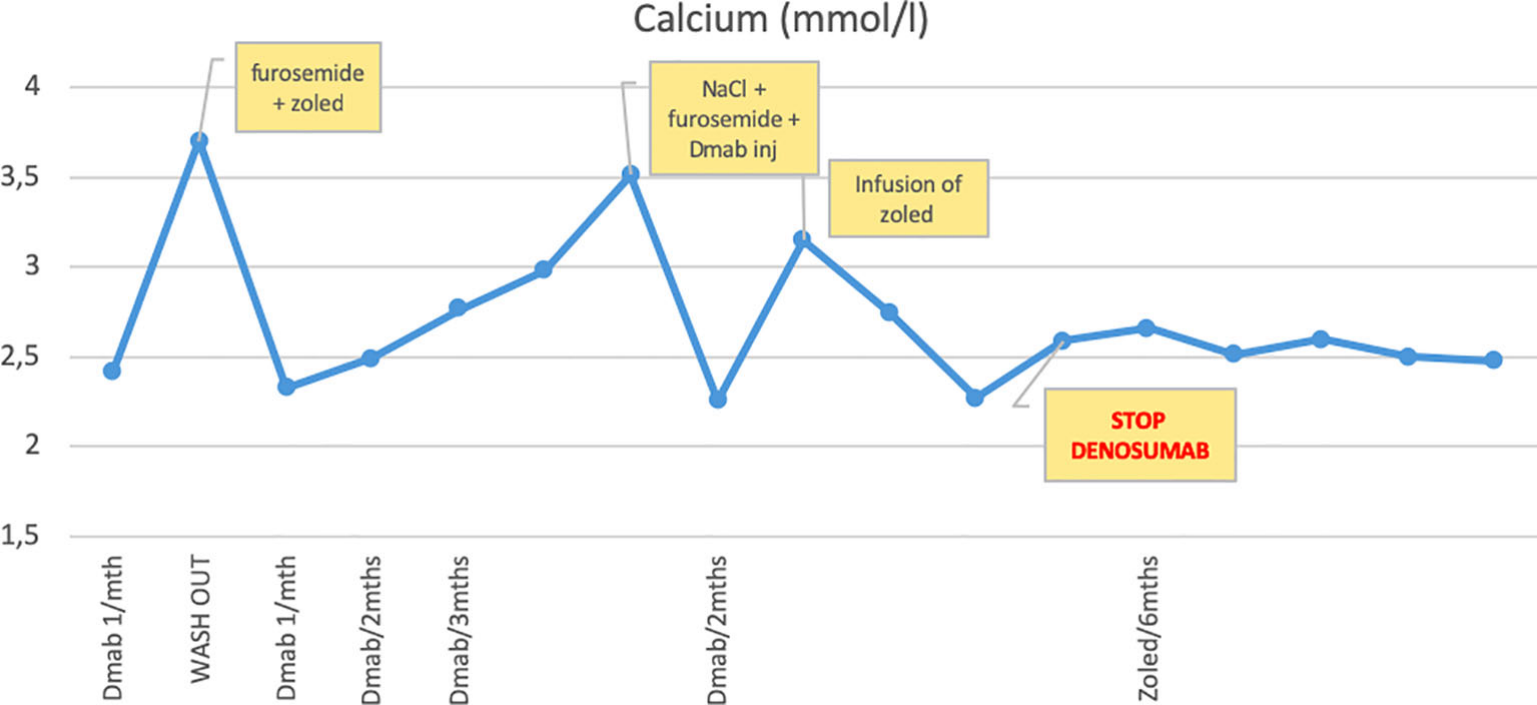
Trend of serum calcium levels during denosumab therapy. Hypercalcemia occurring during wash-out period, progressive rise of serum calcium levels when the injections are spaced and calcium levels remained below the upper limit of normal with 6 monthly IV zoledronic acid infusions.

In the **Table 1** we describe the mineral homeostasis during denosumab treatment, with particular focus on the second course of the treatment, while the **Figure 2** shows the trend of serum calcium levels.

### Bone Remodeling, Modeling and Growth During Denosumab Therapy

**Figure 3:**
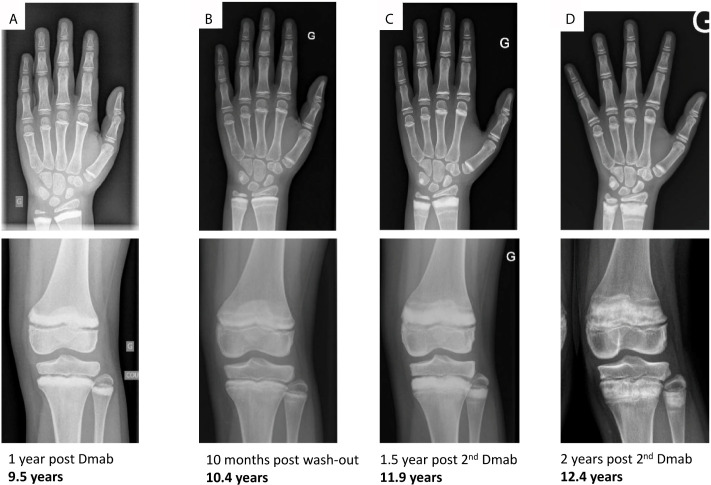
Hand and knee radiographs during follow-up. Radiographs taken **(A)** after 1 year of denosumab (onset of sclerotic metaphyseal bands), **(B)** after 10 months of denosumab wash-out (reduction of sclerotic bands), **(C)** after 1.5 years of the 2^nd^ course of denosumab (more pronounced sclerotic metaphyseal bands) and **(D)** after 2 years of the 2^nd^ course of denosumab (severe and persistent sclerotic metaphyseal bands).

**Figure 4:**
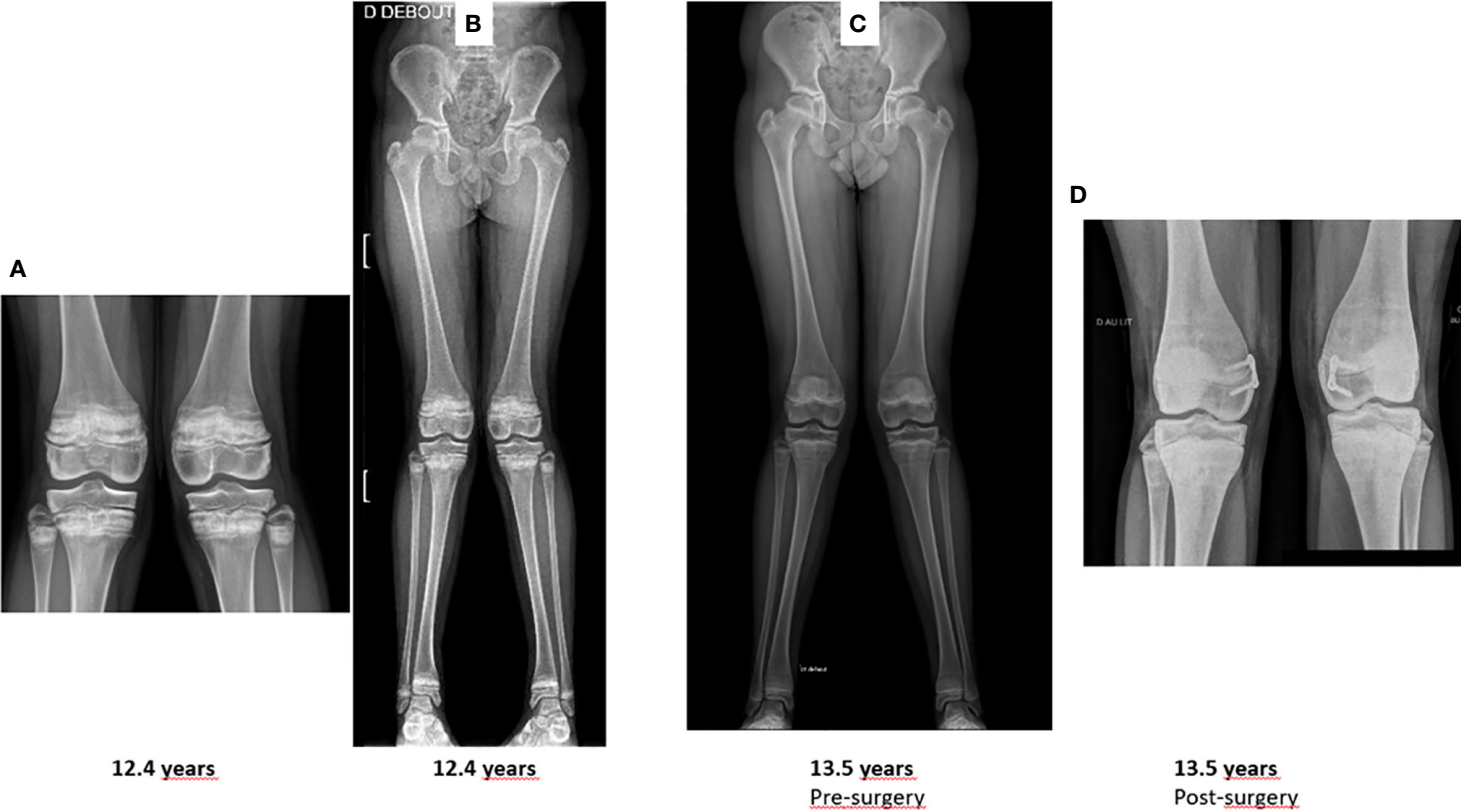
Knees and lower limb radiographs. Lower limbs were imaged standing using EOS. **(A, B)** lower limb deformities at the age of 12.4 years. **(C)** deformities worsened after one additional year, **(D)** post-surgery radiographs of knees.

**Figure 5:**
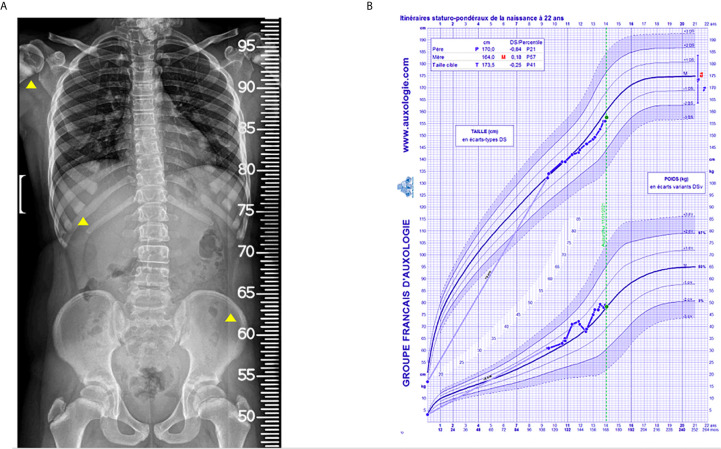
Spine, thoracic and hip radiographic view done by EOS and growth chart. **(A)** The radiograph was done after 2 years of the 2^nd^ course of denosumab showing sclerotic bands at ribs, hips and shoulders. **(B)** Evolution of growth.

In parallel to the rapid changes of mineral homeostasis during denosumab treatment, we observed an amplified bone remodeling in response to denosumab therapy. After 1 year of treatment, the patient developed sclerotic metaphyseal bands visible on radiographs. Sclerotic lines partially vanished during wash-out periods or when the injections of denosumab were spaced (**Figures 3A–D** and **Figures 4B, C**). These bands were more pronounced on long bones, but diffuse osteocondensation of ribs, hips and shoulders was also detected (**Figure 5A**).

**Figure 6:**
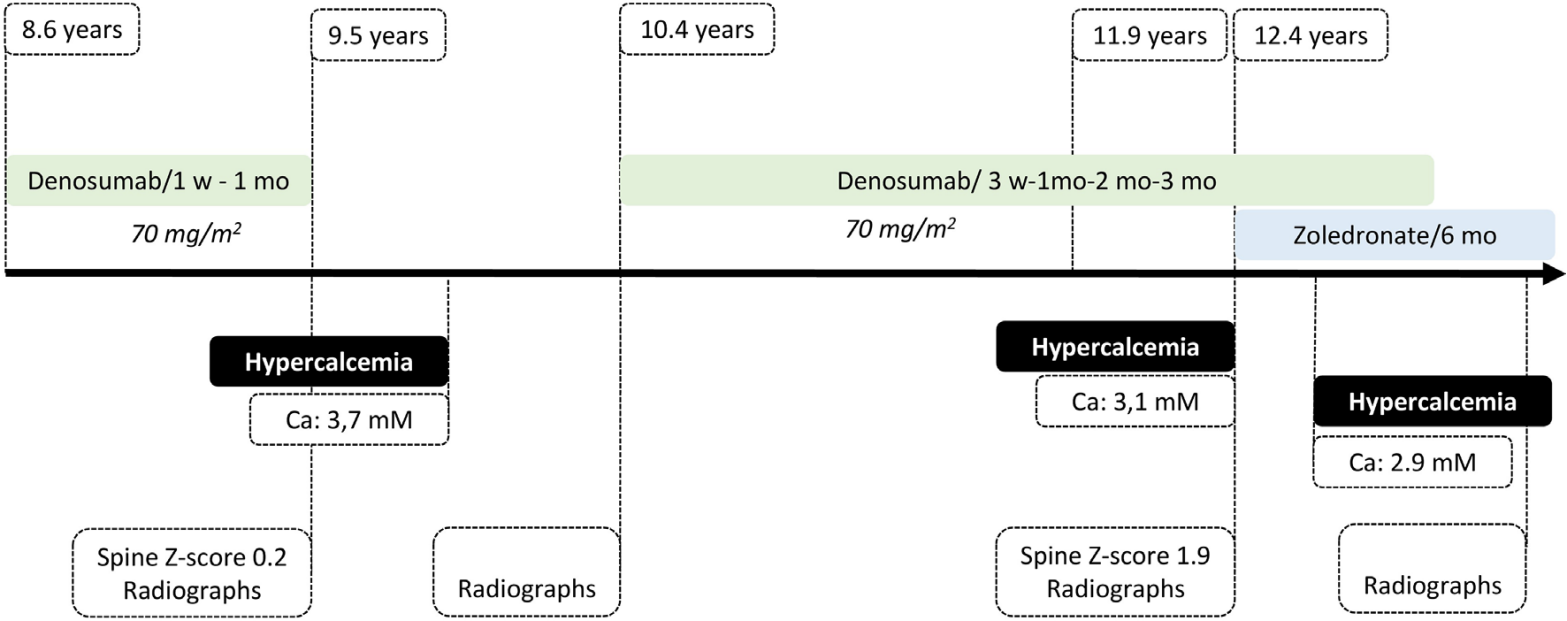
Chart of the patient follow-up.

As expected, the spine bone mineral density measured by DEXA and its relative z-score progressively increased between the age of 9.5 and 12.4 years from 0.841 g/cm^2^ to 1.066 g/cm^2^, and from 0.7 to 1.9, respectively, see **Figure 6**.

During the follow-up we continue monitoring patient’s growth, that was regular and linear until he was 11 years old and 4 months. Then we documented a diminished growth velocity during the period that preceded puberty (**Figure 5B**).

Moreover, it is worth signaling that over the years the patient developed lower limb deformities, i.e. *genu valgum* with intermalleolar distance of about 11 cm (**Figure 4**), which have become progressively more and more painful and eventually required bilateral epiphysiodesis.

## Discussion

A recent review on denosumab therapy for pediatric bone disorders collected reports on 45 children who received denosumab for various conditions (9). This includes children treated with denosumab to target the RANKL/RANK/OPG, signaling pathway involved in the development of ABCs and tumor growth (6).

Lange et al. firstly described the use of denosumab in two boys aged 8 and 11 years to treat recurrent ABCs localized at the C5 vertebrae, and in whom surgery and arterial embolization had failed. Patients received high doses of denosumab, similarly to our report, i.e., 70 mg/m^2^ monthly (Patient 1) 70 mg/m^2^ weekly for 4 weeks and then 70 mg/m^2^ monthly (Patient 2). Both patients recovered from pain and neurological symptoms and showed tumor regression respectively after 2 and 4 months of treatment. During the 2 and 4 months of follow-up, no adverse effects was reported apart from asymptomatic hypocalcemia in one of the patients (7).

Several other cases have been subsequently reported. Doses of denosumab ranged from 1.6 mg/kg monthly to 70 mg/m^2^ monthly for a duration of 4 months to 2 years. One patient developed transient hypocalcemia during the course of treatment (6, 12, 16–18).

Hypercalcemia has been reported in children upon denosumab therapy on average 5 months after the discontinuation of their treatment (see **Table 2**). In those cases, denosumab was used as an adjuvant therapy in giant cell tumors of bone (19–21), juvenile Paget’s disease (22), fibrous dysplasia (23), and osteogenesis imperfecta type VI (24, 25) or ABCs. In ABCs, children who presented rebound hypercalcemia received doses similar to our current report (13, 14). Many reports did not contain information about serum calcium after or during denosumab treatment; follow-up after denosumab discontinuation was often short likely underestimating the occurrence of mild hypercalcemia. In our experience, severe hypercalcemia occurred after discontinuation of denosumab or when the doses were spaced. It becomes clear that longer follow-up is required to evaluate the real prevalence of this post-discontinuation effect. In adults treated with long-term denosumab therapy, only few cases have been described (26, 27).

**Table 2:** Post-discontinuation hypercalcemia after high dose denosumab in pediatric series.

Reference	Tumor	Patient	Dose	Duration	Response	Post-discontinuation side effects	Timing of hypercalcemia	Total doses
Gossai et al. (19)	Metastatic GCTB of the knee	F 10 yo	120 mg weekly (4 times), 120 mg monthly	24 months	Clinical and radiological improvement	Severe hypercalcemia, metaphyseal bands in long bones (osteopetrosis)	5 months after last denosumab injection	27
Uday et al. (20)	GCTB sacrum sacrum scapula	M 15 yo F 14 yo M 40 yo	120 mg weekly (4 times), 120 mg monthly	3.6 y 1.3 y 4.0 y	Clinical and radiological improvement	Severe hypercalcemia Severe hypercalcemia/ONJ Severe hypercalcemia	7 months after last inj. 6 months after last inj. 5.5 months after last inj.	46 18 51
Setsu et al. (21)	Sacral GCTB	M 10 yo	120 mg monthly	14 months	Clinical and radiological improvement	Severe hypercalcemia	4 months after last denosumab injection	12
Grasemann et al. (22)	JPD	F 7 yo	0.5 mg/kg x 2 dose spaced 6 weeks	6 weeks	Clinical improvement (pain, mobility)	Severe hypercalcemia	7 weeks after second dose	2
Boyce et al. (23)	FD	M 9 yo	1 mg/kg monthly for 3 months-1.25 mg/kg monthly for 3 months-then 1.5 mg/kg monthly	7 months (interrupted for occurring fracture)	Reduction in pain, BMT, tumor growth rate	Severe hypercalcemia	2 months after last denosumab injection	7
Trejo et al. (24)	OI type VI	M 4.6 yo F 3.9 yo	1 mg/kg every 3 months		Increased BMD	Hypercalcemia during the interval between denosumab injections	7 and 12 weeks after the preceding injection	9 6
Hoyer-Kuhn et al. (25)	OI type 1, 3 & 4	10 children 5-11 yo	1 mg/kg every 3 months	12 months	Increased BMD	Mild hypercalcemia	Documented at the end of the trial	4
Kurucu et al. (14)	ABC	9 cases (5 M; 4 F); median age 12.5 yo	70 mg/m^2^ weekly (4 times) then monthly	Median 12 months	Clinical and radiological improvement	Severe hypercalcemia in 2 patients who had received 17 doses of Denosumab	5 months after last inj.	17
Dürr et al. (13)	ABC	6 cases (4 F; 2 M) median age 17 yo	120 mg weekly (4 times), 120 mg monthly / 60mg every 4 weeks with two additional doses on days 8 and 15 in pt 6 yo	Median 12 months	Clinical improvement and radiological stability	Severe hypercalcemia in patient who received 50% of proposed dosage	6 months after last inj.	15
Sydlik et al. (11)	GCTB GCTB ABC ABC	F 12 yo M 13 yo M 12 yo M 6 yo	60 mg on days 1, 8, 15, 28, and then monthly	14 months 14/7 months 17 months 9 months	Clinical and radiological improvement	Severe hypercalcemia	2 m after last inj. 2-3 m after last inj. 1 m after last inj. 3 m after last inj.	14 17/10 17 9
Raux et al. (15)	ABC	M 8 yo M 8 yo (our case, see below)	70 mg/m^2^ weekly (4 times) then monthly	17 months	17 months	3 episodes of hypercalcemia	3, 5, 6 m after last inj.	20
Our case	ABC	8 yo	70 mg/m^2^ weekly (4 times) then monthly 70 mg/m^2^ weekly (4 times) then monthly, then every 2 mon, finally every 3 mon	12 months 30 months	Clinical improvement and radiological stability	Severe hypercalcemia, metaphyseal bands in long bones Severe hypercalcemia, metaphyseal bands in long bones	5 months after last inj. 3 months after last inj.	15 22

GCTB, giant cell tumor of bone; JPD, juvenile Paget's disease; FD, fibrous dysplasia; BMT, bone marker turnover; OI,osteogenesis imperfecta; ABC, aneurysmal bone cyst.

Towards the end of denosumab activity, the rapid recovery of bone resorption and the release of inhibition of osteoclast maturation and action may lead to a rebound hypercalcemia. Children may be particularly at risk for this rebound osteoclastic activity due to their higher baseline bone turnover (9). Rebound may also be more frequent in children with high bone turnover disorders, which usually support the use of denosumab in children (9). Therefore, a gradual tapering of the denosumab injections has been proposed to prevent the development or recurrence of hypercalcemia (13, 14). As we have shown in this report, alternative, or even, additional strategies may be necessary to inhibit osteoclastogenesis and slow the calcium release from the bone. Bisphosphonates are commonly used in adults to prevent rebound hypercalcemia (27–29); they are incorporated into the bone matrix, tampering the osteoclasts activity when denosumab is interrupted. Bisphosphonates should as well be considered in children at high risk of hypercalcemia because of denosumab therapy (11). In our experience, zoledronic acid allowed the rapid decrease of serum calcium and the prevention of recurrent episodes of symptomatic hypercalcemia.

In addition, use of denosumab in young patients whose growth plate have not yet closed, could result in changes similar to osteopetrosis (30). It is reported that in children treated with bisphosphonates, histological analyses of growth plates revealed retention of calcified cartilage, which is hypothesized to represent horizontal trabeculae formed during the temporary inhibition of epiphyseal activity; dense sclerotic bands on radiographs are seen as a consequence (31). Because denosumab and bisphosphonates both inhibit osteoclast function, the same underlying mechanism may explain the observed skeletal effects with denosumab (32), as in our patient. Experience with bisphosphonates in children is more extensive than that of denosumab, and few complications related to the sclerotic lines have been reported. However, the consequences of long-term bone turnover suppression on the growing skeleton have not been determined. To date, there are no reports suggesting significant effects of denosumab on linear growth (32). However, we think that denosumab impacted bone modeling in our case report through the alternance of suppressed and active bone resorption when denosumab was stopped and resumed or when injections were spaced.

On the other hand, growth chart of our patient shows a linear and regular pattern until the period that preceded puberty. This is probably due to the physiological deceleration of growth velocity before puberty, but we can’t exclude that the repeated action of denosumab on growth plates also played a role. It is possible that the inhibition and subsequent rebound osteoclasts activity have damaged growth plates cartilage and impacted linear skeletal growth. Further studies may be useful in this field.

Bone mineral density measured by DEXA and its relative z-score progressively increased during follow-up, as expected with age, height, weight and during pubertal development. The factors that contribute to the pubertal increase in mineralization are not fully known, but a critical role belongs to the sex steroids (33). The concomitant denosumab therapy in our case probably also had a role in the increase of bone mass.

In conclusion, more and more studies revealed the efficacy of denosumab treatment not only in giant cell tumors and aneurysmatic bone cysts but also in several other osteolytic diseases. Recently Raux et al. (15) stressed the importance of multidisciplinary discussion on the role of neoadjuvant denosumab treatment in children harboring ABCs. It remains critical to establish the optimal duration of the treatment and at the same time to investigate long term control of tumor growth when surgery is not performed. Moreover, unlike adults, severe hypercalcemia at discontinuation of treatment seems rather frequent in children. It is therefore critical to be aware of strategies for treatment or prevention. In addition, experts’ panels may bring insight for the management of these rare situations and should be easily solicited, as we did for our patient.

This case report has some limitations, such as the lack of long-term follow-up. Even if previously reported in other studies and case series, our aim was to describe detailed perturbations of mineral metabolism during and after denosumab treatment in children and adolescents, and to stress the importance of monitoring systematically growth, limb deformities, serum calcium and mineral homeostasis in children and adolescents receiving denosumab.

## Data Availability Statement

The original contributions presented in the study are included in the article/supplementary material. Further inquiries can be directed to the corresponding author.

## Ethics Statement

Written informed consent was obtained from the minor(s)’ legal guardian/next of kin for the publication of any potentially identifiable images or data included in this article.

## Author Contributions

LB, PB, ET, AL, and PW performed clinical diagnosis and conducted patient management and follow-up. GD and AL drafted the manuscript. LB, PB, ET, GM, PW, and AL performed the critical revision of the manuscript. All authors contributed to the article and approved the submitted version.

## Conflict of Interest

The authors declare that the research was conducted in the absence of any commercial or financial relationships that could be construed as a potential conflict of interest.
